# Transcriptomic Analysis Reveals Candidate Ligand-Receptor Pairs and Signaling Networks Mediating Intercellular Communication between Hair Matrix Cells and Dermal Papilla Cells from Cashmere Goats

**DOI:** 10.3390/cells12121645

**Published:** 2023-06-16

**Authors:** Sen Ma, Dejun Ji, Xiaolong Wang, Yuxin Yang, Yinghua Shi, Yulin Chen

**Affiliations:** 1 College of Animal Science and Technology, Henan Agricultural University, Zhengzhou 450002, China; senma2021@yeah.net; 2 Henan Key Laboratory of Innovation and Utilization of Grassland Resources, Zhengzhou 450002, China; 3 Henan Engineering Research Center for Forage, Zhengzhou 450002, China; 4 Key Laboratory for Animal Genetics and Molecular Breeding of Jiangsu Province, College of Animal Science and Technology, Yangzhou University, Yangzhou 225009, China; djji@yzu.edu.cn; 5 Key Laboratory of Animal Genetics, Breeding and Reproduction of Shaanxi Province, College of Animal Science and Technology, Northwest A&F University, Yangling 712100, China; xiaolongwang@nwafu.edu.cn (X.W.); yangyuxin2002@126.com (Y.Y.)

**Keywords:** hair follicle, hair matrix cells, dermal papilla cells, cashmere goat, ligand-receptor pair, signaling axis, intercellular crosstalk

## Abstract

Hair fiber growth is determined by the spatiotemporally controlled proliferation, differentiation, and apoptosis of hair matrix cells (HMCs) inside the hair follicle (HF); however, dermal papilla cells (DPCs), the cell population surrounded by HMCs, manipulate the above processes via intercellular crosstalk with HMCs. Therefore, exploring how the mutual commutations between the cells are molecularly achieved is vital to understanding the mechanisms underlying hair growth. Here, based on our previous successes in cultivating HMCs and DPCs from cashmere goats, we combined a series of techniques, including in vitro cell coculture, transcriptome sequencing, and bioinformatic analysis, to uncover ligand-receptor pairs and signaling networks mediating intercellular crosstalk. Firstly, we found that direct cellular interaction significantly alters cell cycle distribution patterns and changes the gene expression profiles of both cells at the global level. Next, we constructed the networks of ligand-receptor pairs mediating intercellular autocrine or paracrine crosstalk between the cells. A few pairs, such as LEP-LEPR, IL6-EGFR, RSPO1-LRP6, and ADM-CALCRL, are found to have known or potential roles in hair growth by acting as bridges linking cells. Further, we inferred the signaling axis connecting the cells from transcriptomic data with the advantage of CCCExplorer. Certain pathways, including INHBA-ACVR2A/ACVR2B-ACVR1/ACVR1B-SMAD3, were predicted as the axis mediating the promotive effect of INHBA on hair growth via paracrine crosstalk between DPCs and HMCs. Finally, we verified that LEP-LEPR and IL1A-IL1R1 are pivotal ligand-receptor pairs involved in autocrine and paracrine communication of DPCs and HMCs to DPCs, respectively. Our study provides a comprehensive landscape of intercellular crosstalk between key cell types inside HF at the molecular level, which is helpful for an in-depth understanding of the mechanisms related to hair growth.

## 1. Introduction

Hair fibers are an important class of animal products with exceptional commercial values for certain livestock species, including wool sheep, cashmere goats, rabbits, and others [1]. These fibers are generally yielded from the exquisite and structurally complex mini-organ called the hair follicle (HF) in skin tissues [2]. The elongation and shedding of hair fibers tightly synchronize with the cyclically fluctuating activity of HF [3]. Typically, a complete hair cycling bout is partitioned into three successional phases: anagen, catagen, and telogen [4]. Hair growth mainly occurs in anagen, an active growth stage featured by rapid proliferation and terminal differentiation of hair matrix cells (HMCs) to hair shaft-forming cells [5]. In catagen, the spatiotemporally controlled cessation of proliferation and initiation of programmed cell apoptosis of HMCs cooccur with the gradual termination of hair fiber [6]. The telogen represents a resting stage, in which the fibers completely stop growing and HF keeps in a relatively quiescent state [7].

Although the rhythmic cellular activities of HMCs at distinct stages of hair cycling are directly associated with hair regrowth, dermal papilla cells (DPCs), a specialized fibroblast population surrounded by HMCs, are capable of indirectly manipulating fiber growth via secreting growth factors to modulate the status of HMCs [8]. For example, Telerman et al. found that targeted knockout of B-lymphocyte-induced maturation protein 1 (*Blimp1*) in DPCs causes lowered expression levels of growth factors belonging to the fibroblast growth factor (FGF) and other families, with a significant reduction of proliferating HMCs and a shortened hair phenotype in mice [9]. On the other hand, various signals emanating from HMCs are important for maintaining the identity and function of DP via regulating gene expression. For instance, loss of HMCs-derived sonic hedgehog (Shh) signal resulted in noticeable suppressed expression of known growth factors (e.g., *FGF7/10*, *Noggin*) related to HF growth in DPCs and a decreased proliferation ratio of HMCs [10,11]. Taken together, the above literature strongly hinted that intimate cellular communication between DPCs and HMCs is indispensable for them to execute their individual functionalities in HF cycling and hair growth.

Numerous studies have demonstrated that the specific binding of ligands to their cognate membrane receptors is the main manner mediating the paracrine signaling crosstalk between DPCs and HMCs [12,13,14,15]. Various members of growth factor families, including Wnt, SHH, BMP, FGF, and others, have been identified as the main ligands secreted by both cells, and their cognate receptors are exclusively expressed in corresponding cell types [4,5]. FGF7/10 are known DPC-sourced growth factors with potent hair stimulatory effects, and their cognate receptor FGFR2 is uniquely situated in HMCs [15]. The FGF7/10-FGFR2 ligand-receptor pairs-mediated paracrine crosstalk of DPCs and HMCs is essential for normal HF growth because the dysfunction of *FGFR2* causes anomalies of the hair phenotype and HF pattern in mouse skin [16]. *Shh* is a ligand-encoding gene expressed in HMCs in a cell-specific manner, and its receptor gene, *Ptch*, belongs to one of the signature genes in DPCs [15]. Lack of Shh signals leads to the arrest of HF development and a change in the spatiotemporal expression pattern of cyclin D1, a gene induced by Shh signals to regulate cell growth and proliferation [17]. In addition, the expression levels of the ligands and receptors are controlled by intracellular signaling proteins and nuclear transcription factors (TFs). Inactivation of β-catenin, the key TF in the Wnt pathway, in DPCs resulted in dropped abundances of growth factors such as *FGF7/10* and *BMP6*, reduced proliferation of HMCs, and inhibited hair growth [18]. Vitalization of Wnt signaling via inhibiting GSK-3β, the cytoplasmic enzyme that determines the phosphorylation status of β-catenin, promoted hair growth [19]. Although few studies have constructed the ligand-receptor-cytoplasmic signaling protein-TF axis-mediated intercellular communication channel between DPCs and HMCs [5,15], a systematic understanding of how cellular crosstalk is molecularly achieved is still lacking.

In recent years, the appearance of several ligand, receptor, and TF databases has enabled the discovery of these molecules expressed by a certain cell type or tissue at the global level and greatly facilitated the construction of ligand-receptor pairs mediating intercellular communication via paracrine or autocrine fashion in heterogeneous and homogeneous cell populations from bulk or single-cell sequencing data [20]. For example, Shao et al. developed CellTalkDB, a manually curated database of ligand–receptor interactions for humans and mice, which is widely adopted by researchers to forecast ligand-receptor pair-connected cellular crosstalk of different cell types in tumor niches or complex tissues [21,22]. Moreover, several tools have been devised to infer extracellular-to-intracellular signaling cascades composed of ligand, receptor, cytoplasmic signaling protein, and nuclear TF between cells or tissues from multiple data sources. Choi et al. developed and utilized CCCExplorer to characterize paracrine/autocrine crosstalk of key cell types in non-small cell lung cancer tissue and found that the IL6-IL6R-STAT3 axis mediates the promotive effect of macrophages on tumor epithelial cells [23]. Altogether, these studies proved the very usefulness of similar strategies and tools in deeper understanding the mechanisms related to cell-cell crosstalk and the resultant outcomes in various fields of biology. 

In HF biology, a few studies have exhibited the promising application of the above methods in exploring the signaling cascade mediating intercellular crosstalk of multiple follicular cell lineages. For example, Rezza et al. defined the signature genes of the main follicular cell types and established a ligand-receptor interaction network (including Fgf-Fgfr2/3, Eda-Edar, and Pdgfa-Pdgfra) of DPCs and HMCs, providing an unprecedented understanding of cellular niche crosstalk in mouse skin [12]. However, few studies were performed on fiber-producing livestock, such as cashmere goats and sheep, partially because of the high cost of single-cell sequencing-based data acquisition. Here, based on our previous successes in cultivating DPCs and HMCs from cashmere goat [24,25], we simulated the in vivo interaction status of the cells by utilizing a Transwell-based direct in vitro cell coculture system. Subsequently, we performed economical bulk transcriptomic sequencing of cocultured and monocultured cells and inferred ligand-receptor interaction pairs and signaling axes mediating paracrine or autocrine cellular crosstalk of DPCs and HMCs using CellTalkDB and CCCExplorer [21,23], respectively. Our study identified a series of ligand-receptor pairs and extracellular-to-intracellular signaling cascades responsible for intercellular communication among the cell types and exhibited a successful strategy that explores follicular cell crosstalk in a cost-effective manner.

## 2. Materials and Methods

### 2.1. Farm Animals and Chemical Reagents

Three healthy female Shanbei white cashmere goats (~35 kg, ~3 years old) with independent maternal lineage were chosen as experimental animals from a private farm located in Yangling District, Shaanxi, China (34°28′ N and 108°07′ E). The rearing and management of the goats were performed under the recommended regional guideline (Code: DB61/T 584-2013). The producers of all chemical reagents and their detailed information were listed as follows: fetal bovine serum (FBS; Biological Industries, Kibbutz Beit-Haemek, Israel); Dulbecco’s Modified Eagle Medium:F-12 (DMEM/F-12), Coating Matrix Kit (Thermo Fisher Scientific, Waltham, MA, USA); cDNA synthesis kit, insulin, cholera toxin, epidermal growth factor (EGF), and hydrocortisone (Sigma-Aldrich, Shanghai, China); Roswell Park Memorial Institute (RPMI) 1640 medium without calcium (Genom, Hangzhou, China); Cell Cycle Analysis Kit, Hoechst 33,258, JAK inhibitor (WP1066), recombinant IL1A and leptin (Beyotime, Beijing, China); Chelex 100, penicillin, and streptomycin (Solarbio, Beijing, China); ultraRNA pure kit (Cwbio, Beijing, China); RealStar Green Fast Mixture (Genstar, Beijing, China). Other routine reagents were previously preserved in our labs. All experimental procedures related to animals were approved by the Experimental Animal Manage Committee of Northwest A&F University (Approval ID: 2013-31101684).

### 2.2. Isolation, Mono-Culture and Co-Culture of Follicular Cells from Cashmere Goats

Isolation and culture of primary DPCs and HMCs from the skin tissues of cashmere goats were carried out as we previously did [24,25]. Both cells at the third passage were used in the present study. For cell coculture, HMCs were seeded into the bottom chamber of Transwell dishes coated with coating matrix, and DPCs were seeded into the upper chamber of coculture dishes (*n* = 3). Then, both types of cells were cultivated individually until they reached 50% confluency. After nearly two days, the upper and bottom chambers of Transwell dishes were assembled, and at the same time, the culture medium was replaced with RPMI 1640 with the same supplements as the initial medium. All cells were harvested for cell cycle analysis and transcriptome sequencing after a two-day coculture.

### 2.3. Transcriptome Sequencing

Total RNA was extracted from all samples using the ultraRNA pure kit according to the manufacturer’s guidelines. After quality examination and concentration determination of extracted nucleotides, a sum of 3 µg RNA per sample was used as input for the downstream analysis. Twelve sequencing libraries were generated using the NEBNext^®^ Ultra™RNA Library Prep Kit for Illumina^®^ (NEB, Ipswich, MA, USA) following the manufacturer’s recommendations, and the index codes were added to attribute sequences to each sample. Briefly, mRNA was purified from total RNA using poly-T oligo-attached magnetic beads. Fragmentation was carried out using divalent cations at elevated temperatures in the NEBNext First Strand Synthesis Reaction Buffer (5X). The first strand of cDNA was synthesized using a random hexamer primer and M-MuLV Reverse Transcriptase (RNase H^−^). Second-strand cDNA synthesis was subsequently performed using DNA Polymerase I and RNase H. The remaining overhangs were converted into blunt ends via exonuclease/polymerase. After adenylation of the 3’ ends of DNA fragments, NEBNext Adaptor with a hairpin loop were ligated to prepare for hybridization. In order to select cDNA fragments of preferentially 150~200 bp in length, the library fragments were purified with AMPure XP system (Beckman Coulter, Beverly, MA, USA). Then 3 µL USER Enzyme (NEB, USA) was used with size-selected, adaptor-ligated cDNA at 37 °C for 15 min followed by 5 min at 95 °C before PCR. Then PCR was performed with Phusion High-Fidelity DNA polymerase, Universal PCR primer, and Index (X) Primer. At last, PCR products were purified (using the AMPure XP system), and library quality was evaluated on the Agilent Bioanalyzer 2100 system. The clustering of the index-coded samples was performed on a cBot Cluster Generation System using TruSeq PE Cluster Kit v3-cBot-HS (Illumina) according to the manufacturer’s instructions. After cluster generation, the library preparations were sequenced on an Illumina Hiseq platform, and 125 bp/150 bp paired-end reads were generated. Raw data (raw reads) in fastq format were first processed through in-house perl scripts. In this step, clean data (clean reads) were obtained by removing reads containing adapter, reads containing ploy-N, and low-quality reads from raw data. At the same time, Q20, Q30, and GC content of the clean datawere calculated. 

All the downstream analyses were based on clean, high-quality data. Reference goat genome (code: ARS1.1) and gene annotation files were downloaded from the website (https://www.ncbi.nlm.nih.gov/data-hub/genome/GCF_001704415.2/, accessed on 20 May 2019). The index of the reference genome was built using Bowtie v2.2.3, and paired-end clean reads were aligned to the reference genome using TopHat v2.0.12 [26]. HTSeq v0.6.1 was used to calculate the read counts mapped to each gene [27]. Then Fragments Per Kilobase of transcript sequence per Millions base pairs sequenced (FPKM) value of each gene was calculated based on the length of the gene and the read count mapped. Differential expression analysis of two groups was performed using the DESeq R package (1.18.0) [26]. The resulting *P*-values were adjusted using Benjamini and Hochberg’s approach for minimizing the false discovery rate. Genes with an adjusted *p*-value < 0.05 and |log2(FC)| ≥ 1 found by DESeq were thought to be differentially expressed genes (DEGs). All data were deposited at NCBI (BioProject: PRJNA558436).

### 2.4. GO and KEGG Enrichment Analysis

Gene Ontology (GO) and KEGG pathway enrichment analysis of differentially expressed or selected genes was implemented using g:Profiler (https://biit.cs.ut.ee/gprofiler/gost; version: e109_eg56_p17_1d3191d) [28]. Only the genes with at least one annotation were considered to be part of the g:GOSt domain, and the g:SCS algorithm with a threshold of 0.05 was adopted.

### 2.5. Construction of Ligand-Receptor Pairs and Intercellular Communication Network

We downloaded the human ligand-receptor interaction database from the online website of CellTalkDB (http://tcm.zju.edu.cn/celltalkdb; accessed on 24 November 2020). After screening differentially expressed ligands and receptors between groups, we manually constructed the ligand-receptor pairs and visualized the network using the circlize R package [29]. We further constructed the intercellular communication (autocrine and paracrine) network using CCCExplorer software (version: 1.0) [23], and the parameters were set as mentioned in Table S1.

### 2.6. Cell Viability Assay

Approximately 5000 goat DPCs were seeded in a 96-well cell culture plate per well. After incubation overnight at 37 °C in a humidified incubator with 5% CO_2_/95% air, culture medium was replaced by fresh medium with the addition of recombinant IL1α (0 ng/mL, 1 ng/mL, 10 ng/mL), leptin (0 ng/mL, 100 ng/mL), or JAK inhibitor (WP1066; 2.5 μM). Six replicates were set up for each treatment. Twenty-four hours later, 10 μL of CCK-8 solution was added to each well, and all cells were cultured for an additional 4 h. Then, OD at 450 nm was measured using a multifunction microplate reader.

### 2.7. Flow Cytometry Analysis

All mono- and cocultures were performed as described above; cell samples were enzymatically digested and collected into 1.5 mL tubes. After a 3–5 min centrifuge at 1000× *g*, supernatants were removed and 1 mL of cold PBS was added to resuspend cells. Then, the above procedure was repeated, and 1 mL of cold 70% ethanol was added to resuspend cells. After being fixed at 4 °C for 12 h, cells were centrifuged for 3–5 min at 1000× *g*. Supernatants were removed again, and 1 mL of cold PBS was added to resuspend cells. A centrifuge was performed in the same condition. Then, 0.5 mL of PI solution was slowly added to tubes for resuspending cells, and the cells were kept at 37 °C for thirty min in a dark room. Finally, all samples were analyzed using flow cytometry (Becton, Dickinson, and Company, East Rutherford, NJ, USA). All experiments were performed in triplicate to assure the reproducibility of the results.

### 2.8. Quantitative Real-Time PCR (qRT-PCR)

The total RNA of all cell samples was extracted using the ultraRNA pure kit (CWBio, Beijing, China) according to the manufacturer’s instructions. The quality and quantity of extracted RNA were assessed with electrophoresis or an ultra-micro spectrophotometer, Nanodrop 2000. A sum of 2 ug total RNA was used in the reverse transcription reaction with a cDNA synthesis kit (Thermo Fisher Scientific, MA, USA). Quantitative real-time PCR analysis was performed in triplicates with the Bio-Rad IQ5 Real-Time PCR system using RealStar Green Fast Mixture (Genstar, Beijing, China) according to the manufacturer’s protocol. Relative abundances of mRNA were determined by the classical 2^−ΔΔCt^ method. *GAPDH* was set as the internal control in all experiments. Information on genes of interest and their primers is detailed in Table S2.

### 2.9. Hocheste 33,332 Staining

Goat DPCs were seeded into a 24-well plate at a quantity of 20,000 cells per well. After overnight incubation, culture medium was replaced by medium supplemented with leptin (100 ng/mL), JAK inhibitor (2.5 μM), or leptin (100 ng/mL) and JAK inhibitor (2.5 μM). The original culture medium was used as a control. Then, the culture medium was removed after 24 h, and 200 μL of Hocheste 33,332 solution was added to each well. After staining for 30 min, liquid was removed again, and 250 μL of PBS was added to wash the well three times. Finally, the staining photos were recorded with an inverted fluorescence microscope. Three replicates were set for each treatment, and five visual fields were randomly selected and recorded for each well.

### 2.10. Statistical Analysis

A two-tailed *t*-test was chosen for the comparison between two groups, and a one-way ANOVA was selected for multiple comparisons among three or more groups (software: GraphPad Prism 8.0.1). All data were represented as mean ± standard deviation (sd). The standard for statistical significance was *p* < 0.05, and the statistical extreme significance was *p* < 0.01. Pearson’s correlation analysis was performed using R [30]. 

## 3. Results

### 3.1. Coculture Altered the Cell Cycle Patterns of Goat DPCs and HMCs

**Figure 1:**
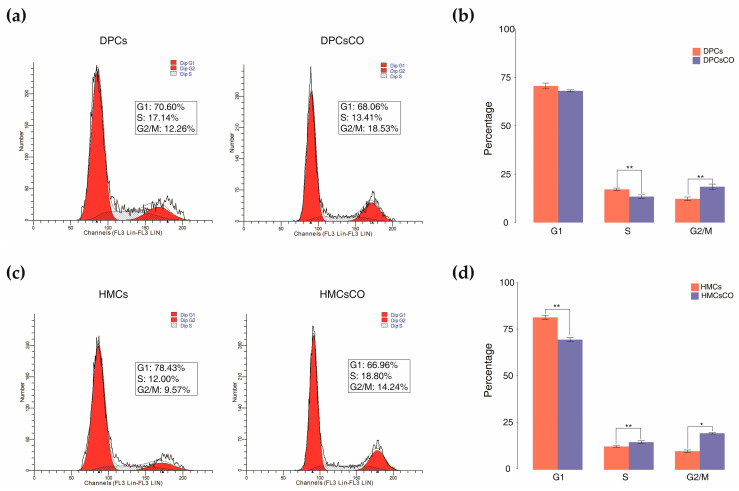
Coculture alters the cell cycle distribution pattern of goat DPCs and HMCs. (**a**,**b**) Representative figures of flow cytometry analysis and the percentages of G1, S, and G2/M phases in the cell cycle of monocultured and cocultured DPCs. (**c**,**d**) Representative figures of flow cytometry analysis and the percentages of three phases in the cell cycle of monocultured and cocultured HMCs. Results shown are the mean ± sd of three replicates in each group. DPCs, dermal papilla cells; HMCs, hair matrix cells; DPCsCO, dermal papilla cells cocultured; HMCsCO, hair matrix cells cocultured. Statistical significance between two groups was determined by a two-tailed student’s *t*-test: * *p* < 0.05, ** *p* < 0.01.

To examine the alterations in cellular status under mono- and cocultured conditions, we performed the cell cycle analysis of goat DPCs and HMCs. As shown in Figure 1a,b, the percentage of cells in S phase is significantly lower in goat DPCsCO compared with DPCs (13.41% vs. 17.14%; *p* < 0.01). Whereas, the proportion of DPC population in G2/M is markedly higher in goat DPCs than DPCsCO (14.24% vs. 9.57%; *p* < 0.01). Similarly, the ratios of goat HMCsCO in the G1 phase dramatically declined when compared with monocultured HMCs (66.96% vs. 78.43%; *p* < 0.01). In contrast, the percentages of HMCsCO in the S and G2/M phases are observably elevated in comparison with individually cultivated goat HMCs (18.80% vs. 12.00%, *p* < 0.01; 14.24% vs. 9.57%, *p* < 0.05). The above results indicated that the goat DPCs and HMCs are capable of mutually affecting the cellular status of the other in vitro culture conditions.

### 3.2. Coculture Changed the Transcriptomic Profiles of Goat DPCs and HMCs

**Figure 2:**
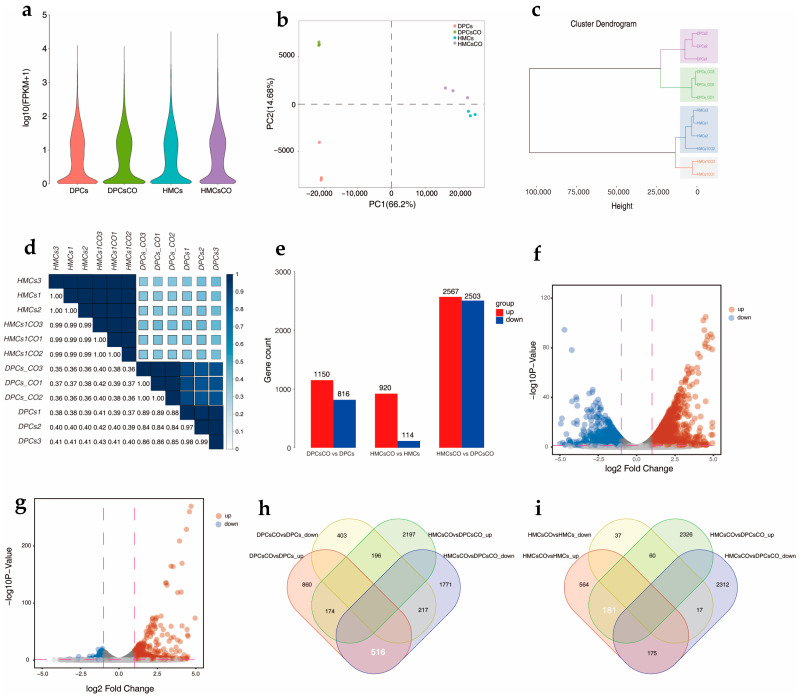
Comprehensive analysis of the transcriptomic profiles of monocultured and cocultured goat cells at a global level. (**a**) Violin plot showing the distribution of transcript abundances measured by FPKM value in four groups; (**b**–**d**) Principal component analysis (PCA), sample clustering analysis, and Pearson’s correlation analysis of four groups using transcriptome data; (**e**) Boxplots showing the counts of differentially expressed genes (DEGs) in three groups; (**f**,**g**) Volcano plot displaying the status of gene expression in DPCsCO versus DPCs and HMCsCO versus HMCs, respectively; (**h**,**i**) Veen graphs showing the identification of 516 and 181 signature genes of DPCs and HMCs, respectively. Data was analyzed from three biological replicates in each group.

To further explore the impact of cell coculture on the transcriptional status of goat DPCs and HMCs, we analyzed the transcriptomic profiles of these cells at a genome-wide level using RNA-seq. As results, 27,492,137~47,450,248 (on average 35,903,531) clean reads and 4.04~6.98 (on average 5.39) G clean bases with qualified error rate, Q20 score, and other standards were acquired from 12 sequencing libraries (*n* = 3 for each group; Table S3). After reads mapping and quantification of the transcripts’ abundances with FPKM, we compared the abundances of genes at a global level among four groups and found a similar pattern emerge across samples (Figure 2a). To further visualize the differences in gene expression among all samples, we performed principal component analysis (PCA) and clustering analysis using the FPKM values of all genes. PCA results showed that goat DPCs/DPCsCO and HMCs/HMCsCO were clearly separated at the first dimension (PC1) of the 2D map, indicating these cells possess a unique set of signature genes pertaining to their cellular identity. In addition, goat HMCs and HMCsCO are situated in adjacent areas on the plot; however, goat DPCs and DPCsCO are visually located in two distant sectors in the second dimension (Figure 2b). These results are highly consistent with the sample clustering analysis, in which four minor clades and two major clades emerged in the plot (Figure 2c). We also performed Pearson’s correlation analysis to quantify the similarity of transcriptomic data among samples at a global level. As expected, the correlation coefficients (0.35~0.41) were lowest between DPCs/DPCsCO and HMCs/HMCsCO (Figure 2d), which fits the very fact that they belong to distinct cell types with differential functions and destiny [5,8]. We also found the correlation coefficients between DPCs and DPCsCO (0.84~0.89) are lower than those between HMCs and HMCsCO (0.99~1.00). This finding suggested that the extent of the fluctuation in transcriptomic profiles of DPCs after coculture is more drastic than that of HMCs. Collectively, the above results demonstrated that goat DPCs and HMCs possess characteristic transcriptomic profiles, and cellular coculture induces remarkable alterations of the holistic gene expression profile in goat DPCs compared to HMCs.

To further probe the detailed changes in cellular transcriptomes, we identified the differentially expressed genes (DEGs) between four sample sets using DESeq2 [26]. As results, a total of 1966 DEGs, including 1150 upregulated DEGs and 816 downregulated DEGs, were identified in DPCsCO compared with DPCs (|log2FC| ≥ 1, p-adjusted value ≤ 0.05, Figure 2e,f). At the same time, a sum of 1034 DEGs, comprising 920 upregulated DEGs and 114 downregulated DEGs, was found between HMCsCO and HMCsv (Figure 2f). In addition, we defined the signature genes of individual cell types by comparing the transcriptomic profiles of HMCsCO and DPCsCO, which are more closely related to their in vivo status. We collectively identified 2567 and 2503 signature genes for HMCsCO and DPCsCO, respectively. The relatively fewer quantity of identified DEGs between mono- and cocultured HMCs in comparison with DPCs clearly implies that the extent of transcriptomic fluctuation at the global level is greater in DPCs than HMCs. These findings are also in high accordance with PCA, sample clustering, and Pearson’s correlation analysis mentioned above. Detailed information on the DEGs identified in each group is provided in Table S4.

Next, we separately performed functional enrichment of upregulated and downregulated DEGs screened in three groups. Several outstanding signaling pathways, including cell cycle, cellular senescence, the TNF signaling pathway, and the p53 signaling pathway, were significantly enriched in upregulated DEGs of DPCsCO. At the same time, many cell cycle regulation-related GO terms, including cell cycle and cell cycle process, are on the list of top-enriched terms. For downregulated DEGs in DPCs, GO terms closely related to developmental modulation or signal transduction emerged. As for HMCs, signaling pathways, including Hippo signaling pathway—multiple species, focal adhesion, oxytocin signaling pathway, and FoxO signaling pathway, were enriched for upregulated genes in HMCsCO. Small molecules (e.g., ion, protein, nucleotide) binding and enzymes (e.g., phosphotransferase, kinase) activity represent the significantly enriched GO terms for upregulated DEGs in HMCs. In addition, we also identified pathways and GO terms related to cellular features of goat HMCs and DPCs. Signaling pathways, including pathways in axon guidance, Hippo signaling pathways, and pathways in cancer, are enriched for upregulated DEGs in HMCsCO compared with DPCsCO. The majority of top-ranked GO terms are related to cell organization (e.g., cell periphery, biological adhesion) and organismal development regulation (e.g., anatomical structure development, cell differentiation). Moreover, a large body of terms involving skin and epithelial development and cellular communication were found. For the overexpressed genes in DPCsCO, their enriched pathways comprise focal adhesion, proteoglycans in cancer, ECM-receptor interaction, the PI3K-Akt signaling pathway, and others. Some GO terms concerning animal development (e.g., anatomical structure development) and regulation of cellular signaling transduction (e.g., regulation of signal transduction, regulation of cell communication) are among the top-ranked terms on the list. Detailed information was provided in Table S5.

Finally, we narrowed down the spectrum of functional signature genes by intersecting the upregulated DEGs among the three groups. As shown in Figure 2h,i, a total of 516 and 181 genes were obtained. A catalog of genes with well-characterized functions, including WNT5A, ESR1, FGF7, and FGF10, are on the list of DPCs. However, fewer genes (e.g., VEGFA) with well-known roles in hair growth were discovered in HMCs. A full list of these genes is provided in Table S6.

### 3.3. Unique Expression Patterns of Genes Related to Hair Composition, Cell Apoptosis, DNA Methylation, and Prostaglandin Metabolism

**Figure 3:**
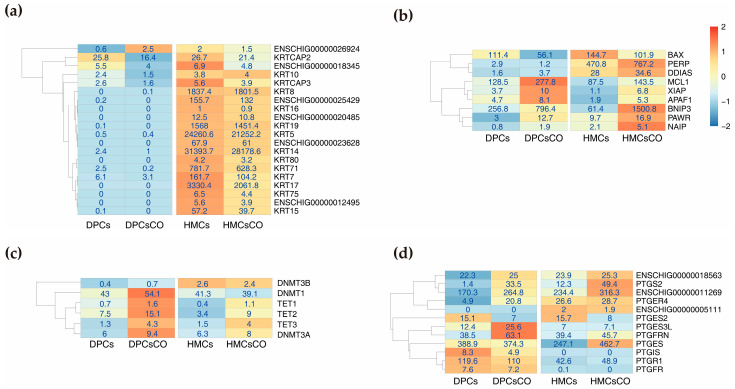
Expression patterns of genes involved in key biological processes in hair growth. (**a**) Heatmap showing the expression patterns of keratins (KRTs) related to hair composition; (**b**) Heatmap of the expression pattern of genes regulating cellular apoptosis in cells; (**c**) Heatmap displaying the relative abundances of genes encoding DNA methylases and demethylases; (**d**) Heatmap characterizing the relative expressions of genes encoding enzymes, receptors, and inhibitors related to prostaglandin synthesis and signaling transduction.

Keratins (KRTs) and keratin-associated proteins (KAPs) are the main structural components of epithelial cells [1]. Thus, we checked the abundances of the genes encoding these proteins and detected a total of 20 genes (FPKM ≥ 1) expressed in cells (Figure 3a). The majority of these genes are exclusively expressed in goat HMCs and HMCsCO. The genes with the highest expression levels include KRT14, KRT5, KRT17, and KRT8, which should be the potential cellular markers of HMCs. The appearance of cell apoptosis events is a pivotal hallmark of the cyclic termination of hair growth [3]. Thus, we focused on the expression of genes encoding apoptosis regulators in individual cell types (Figure 3b). The expression levels of anti-apoptotic genes (DDIAS, MCL1, XIAP, and NAIP) are uniformly elevated in goat DPCsCO; whereas, the abundances of pro-apoptotic genes showed inconsistent patterns. For instance, the expressions of Bax and PERP are downregulated; however, the relative levels of BNIP3 and PAWR arose in DPCsCO compared to DPCs. At the same time, we also found concurrent elevations of pro-apoptotic genes (APAF1, BNIP3) and anti-apoptotic genes (XIAP, NAIP) in HMCsCO. The above results indicated that an intricate balance was maintained by the positive and negative apoptosis-related regulators in these cells. 

Next, we examined the dynamics of DNA methylation and demethylation-related genes in mono- and cocultured cells (Figure 3c). Stable expression patterns were found in genes encoding DNA methyltransferases (DNMT1, DNMT3A, and DNMT3B); whereas, the relative abundances of genes encoding DNA demethylases (TET1, 2, and 3) were significantly upregulated in cocultured cells. This result strongly implies that the DNA demethylation process potentially occurs in cocultured cells. Furthermore, we extracted the expression profiles of genes (FPKM ≥ 1) involved in tissue metabolism, synthesis, and cellular receptor binding of prostaglandin—the hormone with key roles in regulating hair growth [31]—from all samples (Figure 3d). Several genes encoding reductases (ENSCHIG00000018563, PTGR1) and synthases (ENSCHIG00000011269, PTGES) are abundantly and stably expressed in four cell types. The transcriptional level of PTGS2, the enzyme catalyzing the initial step of prostaglandin production, is elevated in cells after coculture. Of note, we found that ENSCHIG00000005111 (prostaglandin F synthase 1), which encodes the enzyme responsible for the reduction of prostaglandin F2α from its precursors, is exclusively expressed in HMCs and HMCsCO, and the cognate membrane receptor (PTGFR) is uniquely expressed in DPCs and DPCsCO. In addition, PTGFRN, which encodes a protein that inhibits the binding of prostaglandin F2α to PTGFR, is widely expressed in all cells. The above findings unveiled that these cells are active sites of follicular prostaglandin biosynthesis. Furthermore, goat HMCs are potentially the main sites of prostaglandin F2α production, and DPCs are the target cells that receive the hormone. Moreover, the biological effect of prostaglandin F2α on DPCs could be finely adjusted by the negative regulator, *PDGFRN*, constitutively expressed by both cells.

### 3.4. Identification of Core Ligands, Receptors, and Transcription Factors Affected by Coculture

**Figure 4:**
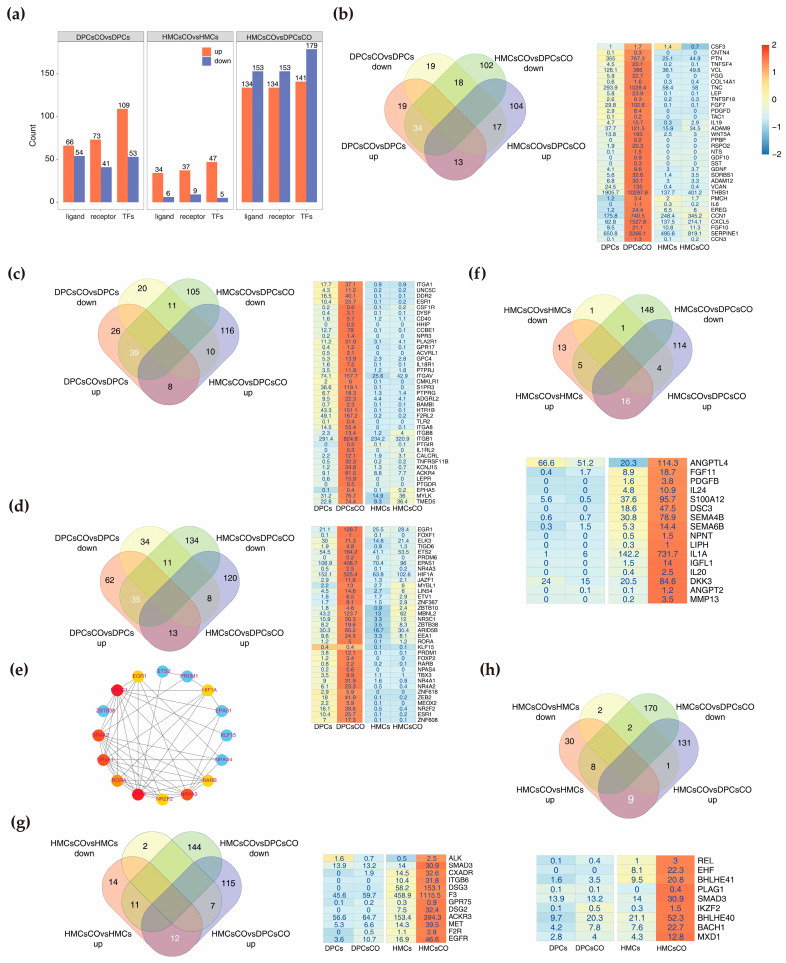
Identification of core ligands, receptors, and transcriptional factors affected by coculture of goat DPCs and HMCs. (**a**) Boxplots showing the counts of ligands, receptors, and transcription factors (TFs) identified from each group. (**b**–**d**) Veen graph and heatmap displaying the key ligands, receptors, and TFs in goat DPCs found by overlapping the differentially expressed genes from DPCsCO versus DPCs and HMCsCO versus DPCsCO; (**e**) Interaction network of TFs detected in (**d**); (**f**–**h**) Veen graph and heatmap showing the key ligands, receptors, and TFs in goat HMCs discovered by overlapping the differentially expressed genes from HMCsCO versus HMCs and HMCsCO versus DPCsCO.

To deeply understand the molecular channels mediating intercellular crosstalk and the mechanisms governing gene transcription regulation, we screened differentially expressed ligands, receptors, and transcription factors (TFs) from DEGs in each group. Ligand and receptor lists (human) were downloaded from the CellTalk Database (http://tcm.zju.edu.cn/celltalkdb/; accessed on 24 November 2020), and TFs lists (human) were downloaded from the Human Transcription Factors website (http://humantfs.ccbr.utoronto.ca/index.php: accessed on 21 May 2020). Gene counts of filtered ligands, receptors, and TFs screened from three combinations are shown in Figure 4a. Next, we screened potential ligands, receptors, and TFs with specific roles in cellular communication via the intersecting results above. A total of 34 ligand-encoding genes (e.g., *WNT5A*, *RSPO2*, *FGF7*, *FGF10*, *LEP*), 39 receptor-encoding genes (e.g., *ESR1*, *ITGA1*, *BAMBI*, *LEPR*), and 35 TF-encoding genes (e.g., *EGR1*, *RORA*, *ESR1*, *PRDM6*) were identified in goat DPCsCO (Figure 4b–d). We also constructed the interactive network of the above TFs and assessed their significance using cyotHubba. As shown in Figure 4e, ESR1, NR4A1/2/3, and NR3A1 are detected as the important nodes in the network. Similarly, a sum of 16 ligand-encoding genes (e.g., *IL1A*, *ANGPT2*, *MMP13*), 12 receptor-encoding genes (e.g., *CXADR*, *EGFR*, *DSG2*, *ITGB6*), and 9 TF-encoding genes (e.g., *REL*, *PLAG1*, *BACH1*, *EHF*) were identified in HMCsCO (Figure 4g,h). These results hinted that these genes may play critical characters in the intercellular communication between DPCs and HMCs and impart transcriptional regulation roles during cellular signal transduction. 

In addition, we also constructed the interaction networks of upregulated TFs in cocultured DPCs and HMCs and elevated their significance in the network using degree scores calculated by cytoHubba [32] (Figure S1). We found that FOS, ESR1, CREB1, HIF1A, and other proteins are among the top-ranked TFs, suggesting their potential crucial roles in DPCs and hair growth. Moreover, two minor networks constituted by DNA methylation process-related TFs (TET1, TET2, and TET3) and circadian rhythm regulation-associated proteins (BHLHE41, BHLHE40, RORA, CLOCK, and CREB1) emerged (Figure S1a). For cocultured HMCs, the top-ranked TFs include NFIL3, CLOCK, and other proteins. At the same time, the circadian rhythm network also appeared (Figure S1b).

### 3.5. Construction of Ligand-Receptor Pairs Mediating the Autocrine and Paracrine Crosstalk between Homogenous and Heterogeneous Cell Types

**Figure 5:**
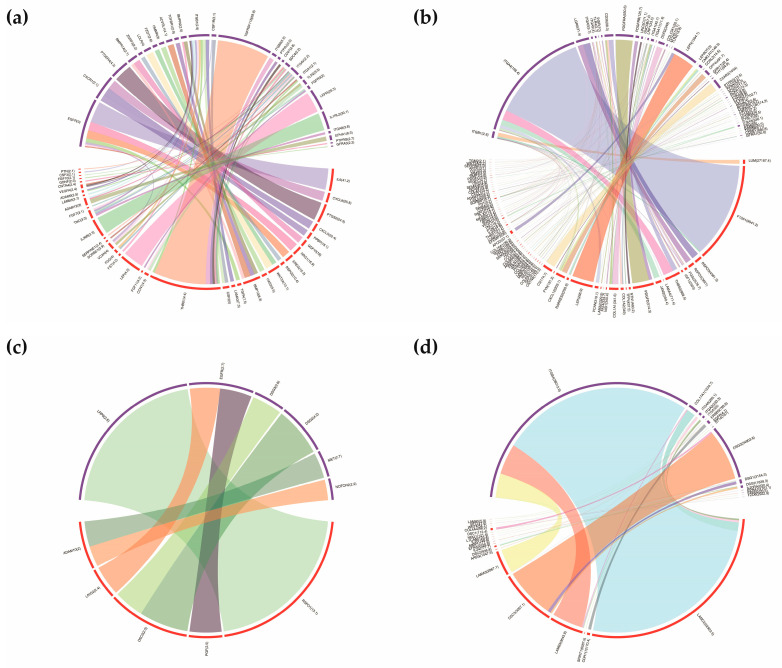
Ligand-receptor pairs mediate the autocrine signaling loop in goat DPCs and HMCs. (**a**,**b**) Circos plots exhibiting the ligand-receptor pairs mediating the autocrine signaling in goat DPCs constructed by differentially expressed ligands and receptors from DPCsCO versus DPCs group and HMCsCO versus DPCsCO, respectively; (**c**,**d**) Circos plots displaying the ligand-receptor pairs mediating the autocrine signaling in goat HMCs built by the differentially expressed ligands and receptors from HMCsCO versus HMCs group and HMCsCO versus DPCsCO, respectively. The width of the edge corresponds to the value of CS.

**Figure 6:**
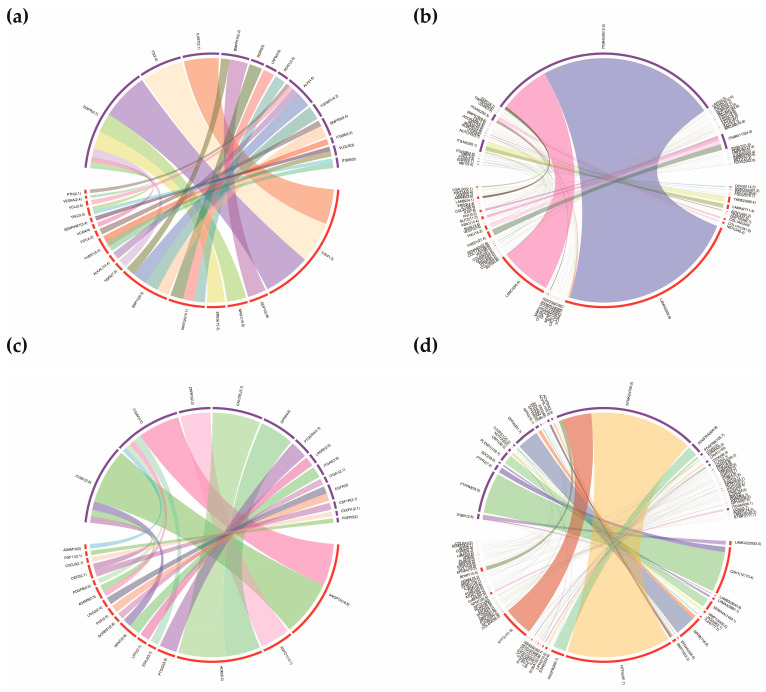
Ligand-receptor pairs mediate the paracrine signaling-linked intercellular crosstalk between goat DPCs and HMCs. (**a**) Ligand-receptor pairs mediating the intercellular communication identified from upregulated ligands in DPCsCO in DPCsCO versus DPCs group and upregulated receptors from HMCsCO versus HMCs group; (**b**) Ligand-receptor pairs mediating signal from DPCs to HMCs identified by upregulated ligands in DPCsCO in HMCsCO versus DPCsCO group, and upregulated receptors in HMCsCO in HMCsCO versus DPCsCO group; (**c**) Ligand-receptor pair mediating signaling from HMCs to DPCs identified by similar strategy in (**a**); (**d**) Ligand-receptor pairs linking signaling from HMCs to DPCs identified by similar strategy in (**b**).

After screening differentially expressed ligands and receptors between three sample sets, we constructed the ligand-receptor pairs mediating the autocrine and paracrine crosstalk among these cells. We utilized two strategies to establish the molecular bridges and quantitatively evaluated the communication score (CS) of the ligand-receptor pair by multiplying the fold change (FC) of the ligand and receptor. For the first strategy, the data was generated by comparing mono- and cocultured cells. As shown in Figure 5, a total of 61 autocrine ligand-receptor pairs, including the top-ranked THBS1-TNFRSF11B, IL6-EGFR, and LEP-LEPR, were found in DPCs. Similarly, seven autocrine pairs, including RSPO1-LRP6 and DSC3-DSG2, were detected in HMCs (Figure 5c). For the paracrine crosstalk from DPCs to HMCs, 26 ligand-receptor pairs, including IL6-EGFR, BMP10-ALK, and EREG-EGFR, were discovered (Figure 6a). In addition, 21 pairs mediating signals from HMCs to DPCs were found (Figure 6c). These pairs comprise ADM-CALCRL, ANGTP2-ITGB1, RSPO1-ZNRF3, and others. 

For the second strategy, the DEGs identified between DPCsCO and HMCsCO were used as input. We identified a sum of 219 autocrine ligand-receptor pairs in goat DPCsCO. The pairs with top-ranked CS included F13A1-ITGA4, LEP-LEPR, PDGFD-PDGFRA, and others (Figure 5b). We also found 166 ligand-receptor pairs constituting an autocrine loop in HMCsCO (Figure 5d). LAMC2-ITGB4, DSC3-DSG3, LAMB3-ITGB4, LAMA4-ITGB4, and others are among the pairs with the highest CS. For paracrine cellular communication from DPCsCO to HMCsCO, a sum of 170 ligand-receptor pairs were screened (Figure 6b). Several pairs, including LAMA2-ITGB4, LAMC3-ITGB4, THBS2-ITGA6, LAMA2-ITGA6, and others, ranked the top among others. We also found 136 ligand-receptor pairs mediating intercellular communication signals from HMCsCO to DPCsCO (Figure 6d). NTF4-NTRK2, NPPB-DPP4, NTF3-NTRK2, CDH1-PTPRM, and others are molecular channels with the highest-ranked scores. Collectively, the above results demonstrated that cellular coculture remarkably affects the patterns of the autocrine signaling loop and the paracrine-mediated intercellular crosstalk between DPCs and HMCs. The above data are provided in Table S7.

### 3.6. Construction of Ligand-Receptor-Signaling Protein-TFs Axis Mediating Autocrine and Paracrine Crosstalk in Cells

**Figure 7:**
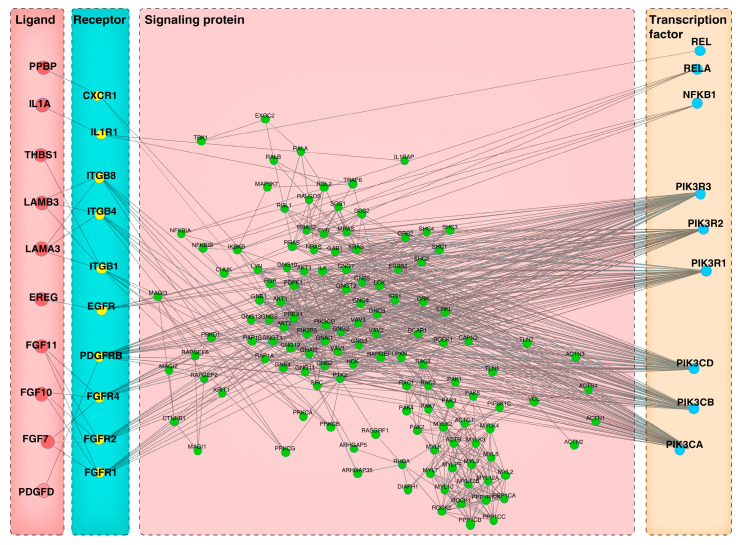
Ligand-receptor-signaling protein-TFs axis mediating the paracrine intercellular communication from goat DPCs to HMCs using differentially expressed genes from DPCsCO versus DPCs and HMCsCO versus HMCs as CCCExplorer input.

**Figure 8:**
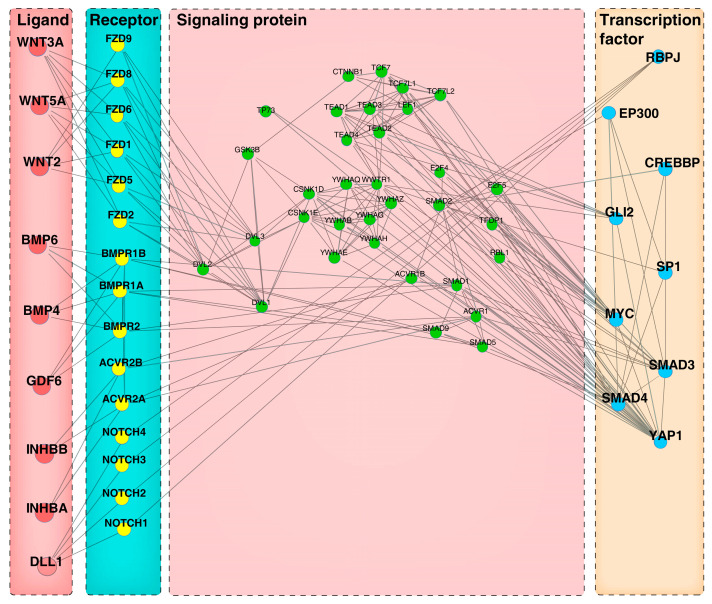
Ligand-receptor-signaling protein-TFs axis mediating the paracrine intercellular communication from goat DPCs to HMCs using differentially expressed genes between DPCsCO versus HMCsCO as CCCExplorer input.

To establish the signaling cascades connecting the intercellular communication between heterogeneous and homogeneous follicular cell types, we used a computational tool-CCCExplorer to delineate the paracrine and autocrine crosstalk networks between DPCs and HMCs. Here, we used two complementary strategies to construct a more comprehensive panorama of cellular communication among the cells: (1) data from the gene expression analysis of DPCsCO versus DPCs and HMCsCO versus HMCs as input, and (2) data generated by bioinformatic analysis between HMCsCO and DPCsCO as input. Through the first strategy, we constructed the signaling network of cellular communication from DPCs to HMCs (Figure 7), which comprises the ligands (e.g., IL1A, EREG, FGF7, FGF10, and PDGFD) with well-characterized functions in hair growth, corresponding membrane receptors (e.g., IL1R1, EGFR, FGFRs, and PDGFRB), cytoplasmic signaling proteins (IL1RAP, MAGI3, CTNNB1, and others), and nuclear TFs (RELA, NFKB1, PIK3Rs, and others). Using the second strategy, we obtained a similar network with valuable information (Figure 8), which contains the TFs (e.g., RBPJ, GLI2, SP1, and YAP1) with explicit roles in the development of skin epidermis and follicular epithelia, the intracellular signaling proteins (e.g., GSK3β, LEF1, and DVL1), membrane receptors (e.g., NOTCH1, BMPR1, and FZD1), and their extracellular binding proteins (e.g., WNT5A, INHBA, and BMP4). Similarly, we constructed two signaling transduction networks linking the transcellular information flow from HMCs to DPCs. We found that several ligands, including PGF, ANGPT2, IL1A, and FGF11, their receptors (e.g., FTL1, TEK, and IL1R1), downstream intracellular proteins (e.g., IL1RA1P), and TFs (e.g., JUND and FOS), are underlined in each network (Figures S2 and S3). The above results indicated that several signaling axes composed of the above components may play important roles in mediating the directional cellular crosstalk between HMCs and DPCs and the intracellular signaling transduction in target cells.

In addition, we also drew the autocrine signaling networks of DPCs and HMCs for each strategy. For instance, an autocrine signaling transduction axis containing FGF7-FGFR1/2/4-IRS1-RELA was established for homogeneous DPCs by utilizing the first strategy. Similarly, the signaling pathways containing IL1A, its cognate receptors IL1R1/2, intracellular proteins (e.g., IL1RAP, TAB1, and MAP3K7), and nuclear TFs (e.g., FOS and JUN) were discovered in HMCs using the same method. Moreover, we also used the second strategy to construct the signaling cascade in the above cells. Detailed networks were shown in Figures S4–S7.

### 3.7. Construction of Ligand-Receptor-Signaling Protein-TFs Axis Mediating Autocrine and Paracrine Crosstalk in Cells

**Figure 9:**
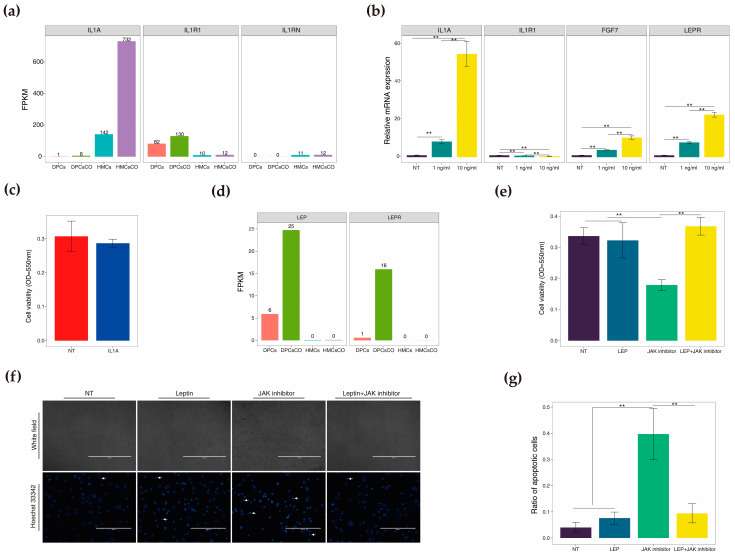
Treatment of goat DPCs using recombinant IL1A and leptin alters cellular status. (**a**) Relative abundances of IL1A, IL1R1, and IL1RN in four cell types; (**b**) Relative mRNA expression of IL1A, IL1R1, FGF7, and LEPR in goat DPCs treated with 0, 1, and 10 ng/mL recombinant IL1A protein (*n* = 3); (**c**) Cell viability of goat DPCs treated with 0 or 10 ng/mL recombinant IL1A protein (*n* = 6); (**d**) Relative abundances of LEP and LEPR in four cell types; (**e**) Cell viability of goat DPCs treated with recombinant leptin protein (0 or 100 ng/mL), JAK inhibitor (WP1066, 2.5 μM), or the combination of recombinant leptin protein (100 ng/mL) and JAK inhibitor (2.5 μM), *n* = 6; (**f**) Cellular apoptosis of goat DPCs in four groups indicated by Hoechst 33,342 staining, with the arrow head displaying the condensed cell nucleus of cells that undergo apoptosis (*n* = 3); (**g**) The ratio of apoptotic cells in four groups measured by the percentage of condensed cell nuclei. **, *p* < 0.01.

To verify the cellular crosstalk information obtained above, we treated the goat DPCs with recombinant proteins to simulate paracrine intercellular communication from HMCs to DPCs and autocrine intracellular crosstalk among DPCs. Our results indicated that IL1A-IL1R1 is the ligand-receptor pair mediating transcellular signal transduction from HMCs to DPCs. At the same time, several studies have revealed that IL1A exerts controversial regulatory roles on hair growth in mammals [33,34]; however, related cellular and molecular mechanisms remain obscure. Firstly, we examined the expression levels of IL1A, its receptor IL1R1, and the antagonist IL1R1-IL1RN in four cell types (Figure 9a). We found that IL1A is almost exclusively expressed in HMCs, and the transcriptional level is significantly boosted after coculture with DPCs (the higher FPKM value of HMCsCO versus HMCs). At the same time, the relative transcript abundance of IL1R1 is lower in HMCs than DPCs; whereas, the antagonist IL1RN is uniquely expressed in HMCs. These data strongly suggest that HMCs are sources of secretory IL1A, and DPCs are the targets of IL1A via specifically binding to its membrane receptor, IL1R1. At the same time, the intensity of IL1A-IL1R1 signaling is finely modulated by the HMCs-originated receptor antagonist IL1RN. We further treated goat DPCs with recombinant IL1A protein and checked the expression of relevant genes and cell viability (Figure 9b,c). We found that the transcriptional levels of IL1A and FGF7, a well-known growth factor excited by IL1A in fibroblasts [35], were significantly improved in treated groups compared to non-treated groups. However, the mRNA expression of IL1R1 was dramatically reduced in the experimental groups compared to the control group, indicating a negative feedback loop may exist. In addition, no significant difference was found in cell viability between the IL1A-treated (10 ng/mL) and control groups. Collectively, the above results hint that IL1A changes the cellular response of goat DPCs at the transcriptional level without affecting cell viability.

Following that, we investigated the effect of LEP-LEPR-mediated autocrine signal vitalization on cultured DPCs. Transcript expressions of LEP and LEPR were unique in goat DPCs and were markedly elevated in cocultured DPCs compared to monocultured cells (Figure 9d). These data clearly imply that LEPR mediates the autocrine influences of LEP on goat DPCs, and the action was intensively strengthened by cellular coculture with HMCs. Past studies have confirmed that JAK mediates the intracellular response of LEP after its binding to the cognate membrane receptor LEPR [36]. Thus, we treated the goat DPCs with recombinant LEP protein, a JAK inhibitor (a cellular apoptosis inducer), and their combination to observe the roles of LEP in goat DPCs (Figure 9e). The cell viability data showed that LEP exerts an insignificant effect on stimulating the growth of DPCs but significantly protects the cells from JAK inhibitor-induced growth inhibition. Moreover, the Hoechst 33,342 staining images displayed a denser pattern of shrunk cell nuclei in the JAK inhibitor group than other groups, as proved by the higher ratio of apoptotic cells (Figure 9f,g). 

## 4. Discussion

In the present study, we provided a comprehensive and explicit landscape of intercellular communication channels between goat follicular DPCs and HMCs by utilizing an in vitro cell coculture tactic to simulate an in-situ crosstalk niche in HF. We identified a series of ligands, receptors, and nuclear TFs substantially affected by cellular coculture and constructed multiple ligand-receptor pairs mediating inter- or intracellular crosstalk in these cells. Importantly, we established complex signal transduction axes-composed networks linking the extracellular signals to intracellular responses at the transcriptional level, exhibiting how cellular crosstalk is molecularly achieved between HMCs and DPCs. In addition, functional validation experiments substantiate the unneglected roles of autocrine and paracrine signals in maintaining the core characteristics of goat DPCs and the very usefulness of the resultant data. 

Firstly, we demonstrated that direct cellular crosstalk obviously changes the cell cycle distribution pattern of both cells, indicating that intercellular signal exchange could directly alter the cellular behaviors of reciprocally interacting cells. Past studies confirmed that spatiotemporally modulated proliferation, differentiation, and programmed apoptosis of the cells inside HF across the entire hair cycle are the cellular foundation of rhythmic hair growth and HF regeneration [13]. Abnormal cell cycle arrest and cell growth inhibition of DPCs caused by androgens (e.g., dihydrotestosterone) are thought to be key factors eliciting androgenetic alopecia [37]. Whereas, the promotive effect of minoxidil, a widely used drug for treating androgenetic alopecia, on the growth of DPCs was considered a potential medical mechanism counteracting the adverse impact of androgen on hair growth [38]. Rapid proliferation and subsequent terminal differentiation of HMCs are the hallmarks of anagen; whereas, the gradual degradation of such activities and the appearance of programmed cell apoptosis hint at the occurrence of catagen [39]. For example, activin B, an effective stimulator of hair growth, boosted the cell cycle progression and proliferation of human HMCs via activating ERK signaling [40]. On the other hand, specific proliferation suppression of HMCs by dihydrotestosterone-inducible IL-6 from DPCs resulted in inhibited human hair growth [41]. The above literature suggests that accelerated cell cycle progression and enhanced cell proliferation of both cell types are positively linked to hair growth, and their disturbances hinder hair elongation. Combined with our analytical results, coculture strengthens the stimulating capacity of DPCs and HMCs in hair growth by driving the occurrence of related events. 

Next, we noticed the intensity of transcriptomic alteration at the genome-wide level is stronger in DPCs than HMCs, as indicated by PCA, Pearson’s correlation, and other analyses. The loss of intrinsic hair-inducing properties of DPCs when cultured in dishes was frequently observed in humans and rats [42,43], which accompanied significant changes in global gene expression profiles. Coincubation of DPCs with skin-derived keratinocytes efficiently maintained the functional characteristics of the cells [42], indicating that keratinocytes are capable of restoring the expression patterns of genes related to hair-inducing capacity in DPCs via keratinocyte-originated cytokines or other factors. In accordance with previous studies, we found that an array of genes (e.g., *WNT5A*, *FGF7*, *FGF10*, and others) closely associated with cellular inductivity are specifically upregulated in DPCsCO, and the expressions of some genes (e.g., *VEGFA*) with well-characterized roles in hair biology are uniquely elevated in HMCsCO. Taken together, in vitro coculture of DPCs and HMCs should be a feasible method to recover or maintain the inherent inducive capacity of animal DPCs, and the present proposal is supported by the varied expression profiles of genes pertaining to hair growth in cells under direct coexistence. 

DNA methylation is an epigenetic and reversible nucleotide modification that is controlled by a set of methyltransferases and demethylases [43]. We showed that the genes encoding enzymes related to maintenance (i.e., *DNMT1*) and de novo methylation (i.e., *DNMT3A* and *3B*) are steadily expressed in mono- and cocultured cells; whereas, the expression levels of genes encoding DNA demethylase (*TET1*, *2*, and *3*) are significantly elevated in cocultured cells. These results strongly hint that cocultured cells undergo an actively regulated DNA demethylation process. Previous studies implied that global DNA methylation levels are higher in the cashmere goat skin tissues from telogen than anagen [44], and alterations of DNA methylation status in coding genes and non-coding genes are associated with the development and pathogenesis of HF [45,46]. Subsequent examination of the global DNA methylation status and identification of affected genes will provide deeper insight into how intercellular communication in HMCs and DPCs is finely tuned at the epigenetic level. 

Prostaglandin F2α (PGF2α) and their analogues have been validated as potent hair growth stimulators via accelerating the conversion of HF from telogen to anagen stage in mice [47]; thus, researchers proposed PGF2α as a promising drug to treat androgenic alopecia and other hair loss-related diseases [48]. However, how PGF2α induces hair growth remains elusive. Our results suggested that PGF2α is the hormone mainly synthesized and excreted by HMCs and received by the target cells, DPCs, through binding to the specifically expressed membrane receptor, PTGFR. The present finding is highly consistent with the human study showing that PGF2α is predominantly produced and secreted by HF-derived keratinocytes but not DPCs or other fibroblasts [31]. The above evidence strongly indicates that the hair growth stimulatory role of PGF2α is achieved by directly acting on the signaling centers of HF, the DPCs. In other tissues and organs, PGF2α was found to vitalize multiple signaling pathways to exert its various biological functions, including ERK1/2, PI3K, and other signaling [49]. Deeper studies are needed to verify which intracellular signaling cascade is activated by PGF2α in DPCs, to clarify underlying mechanisms related to hair growth promotion, and to further support the promising clinical applications in humans.

Further, we discovered a myriad of ligands, receptors, and TFs mediating or influenced by intercellular crosstalk between DPCs and HMCs via intersecting the upregulated genes boosted by cell coculture and signature genes of each cell type. The important characters of partial genes in hair biology have been revealed. For example, Wnt5a, a DPC-specific secreted ligand, is essential for normal differentiation and cell fate decisions in HMCs [50]. HHIP, the inhibitor of Shh signals located on the cell plasma membrane, participates in the precise regulation of the Shh-FGF7/10 axis-mediated signaling loop between DPCs and HMCs [15]. Targeted knockout of the TF Prdm1 in the HF dermis leads to aberrant phenotypes of hair development and a significant reduction of proliferating HMCs [9]. A null mutation of *egfr* in mice resulted in premature differentiation of follicular keratinocytes and hair shaft abnormalities [51]. Inactivation of *rel* impeded HF morphogenesis and caused failure of hair shaft development in a model organism [52]. Although the above cases demonstrated the usefulness of the present strategy in screening candidate genes with crucial roles in hair growth, the molecular functions of a large number of genes remain unknown.

Ligand-receptor binding is one of the most important and prevalent approaches to mediating autocrine or paracrine signaling communication in biology [20]. In the present study, we constructed the ligand-receptor interaction pairs responsible for intercellular crosstalk between DPCs and HMCs. *THBS1* encodes an extracellular matrix glycoprotein with various functions, and its expression is restricted to follicular DPCs [53]. TNFRSF11B was reported to bind THBS1 in human vascular endothelial cells [54], raising the possibility that THBS1-TNFRSF11B constitutes an autocrine signaling axis in DPCs, even though the functions are unknown yet. DSC3-DSG3 was predicted as the ligand-receptor pair mediating autocrine communication among HMCs. In keratinocytes, heterophilic bindings of desmogleins (DSGs) and desmocollins (DSCs) are important for maintaining intercellular adhering junctions and confer structural strength to the epidermis [55]. DSC3 and DSG3 were found to colocalize to the HMCs of human HF at the protein level [56], raising the possibility that DSC3-DSG3 should exert pivotal roles in determining HMC adhesion and hair shaft formation. We also identified a dozen ligand-receptor pairs mediating the paracrine interactions between DPCs and HMCs. DPCs-derived EREG promotes human HF growth and enhances the proliferation and differentiation of HMCs via binding to its receptor EGFR [41], which is consistent with our finding that EGEG-EGFR mediates the signal transmitting from DPCs to HMCs. In addition, we found that RSPO1-ZNRF3-linked HMCs and DPCs intercellular communication is possibly crucial for the maintenance of the hair-inducing capacity of DPCs through the potentiating Wnt signaling pathway. ZNRF3 functions as a transmembrane E3 ubiquitin ligase to ubiquitinate and degrade Wnt receptors and subsequently antagonize Wnt signaling [57]. Whereas, R-spondin proteins are capable of interacting with ZNRF3 and inhibiting its ligase activity, leading to the fortification of Wnt signaling [58]. Taken together, these studies demonstrated that ligand-receptor interaction-mediated autocrine or paracrine signaling between DPCs and HMCs is indispensable in multiple facets of hair biology. Functional validation of more ligand-receptor pairs will be beneficial for an in-depth understanding of the molecular mechanisms governing intercellular crosstalk between DPCs and HMCs and how different cell types coordinate through signaling exchange in HF.

Identification of ligand-receptor-activated intracellular signaling pathways is helpful for understanding how intercellular communication is achieved at the molecular level and how the physiological functions of multiple cell types are spatiotemporally coordinated in a tissue or organ [59]. We utilized CCCExplorer to deduce a ligand-receptor-intracellular signaling protein-TF axis-linked autocrine or paracrine signaling exchange channel in goat DPCs and HMCs. We found that the INHBA-ACVR2A/ACVR2B-ACVR1/ACVR1B-SMAD3 signaling axis mediates intercellular signal transduction from DPCs to HMCs. *INHBA* was determined to be the core signature gene of DPCs [15], and its loss-of-function caused a slower hair growth rate and a shorter hair phenotype in mice [60]. Binding of INHBA to the ACVR2A/ACVR2B and ACVR1/ACVR1B-composed activin receptor complex results in vitalization of the intracellular kinase activity of the complex, and the phosphorylation of Smads to activate gene transcription was recorded in multiple cell lines [61]. Thus, it is reasonable that stimulating the proliferation of HMCs by DPC-produced INHBA via the above signaling axis underlies the roles of INHBA in hair biology because Smad3 is well known to induce cell expansion in several cell types [62,63]. In addition, the construction of several hair growth factors (e.g., FGF7/10, VEGFA, PDGFD, and EREG) related to the extracellular to intracellular signaling transduction axis proposes how such factors participate in hair growth modulation via mediating intercellular crosstalk between DPCs and HMCs.

Finally, we preliminarily verified the physiological functions of the IL1A-IL1R1 and LEP-LEP pairs at the cellular level. Our results exhibited that HMC-derived IL1A significantly improves the expression of the growth factor FGF7, a potent growth factor released by DPCs, indicating IL1A should be a potential hair stimulator. This notion conflicts with the finding that IL1A inhibits the growth of isolated human HF in vitro [33]. Whereas, a recent study showed that IL1A administration accelerates the conversion of mice’s HF from telogen to anagen and promotes hair regrowth via inducing hair follicle stem cell proliferation [64]. Major differences in the conditions of in vitro and in vivo experiments and the stages of hair growth may be possible reasons for the above discrepancy. Leptin was recognized as a hair growth inducer [65], and its knockout caused retardation of anagen initiation in mice [66]. We showed that leptin exerts its biological function in DPCs through a LEP-LEPR-mediated autocrine signaling loop. We also proved that recombinant leptin protects DPCs from JAK inhibitor-induced JAK2 and STAT3 activity suppression, which causes cell growth depression and cell death. Previous studies displayed that leptin exerts its various biological functions via superficially activating the JAK2/STAT3 signaling pathway [36,67]; however, the signal activation could be blocked by all-trans retinoic acid [68], a hair growth depressor specifically targeting DPCs [69]. Taken together, the growth-stimulatory effect of leptin in HF biology could be achieved by counteracting the suppressive roles of hair growth inhibitors in DPCs via activation of JAK2/STAT3 signaling.

## 5. Conclusions

In vitro coculture significantly changes the cell cycle distribution and global gene expression patterns of goat DPCs and HMCs compared with monocultured cells. Transcriptomic analysis uncovers key ligands, receptors, TFs, and biological events affected by cellular interaction. The construction of ligand-receptor pairs and the extracellular-to-intracellular signaling transduction axis provides unprecedented insight into how intercellular communications between DPCs and HMCs are achieved in autocrine or paracrine fashion. Preliminary functional validation of the IL1A-IL1R and LEP-LEPR pairs verifies the usefulness of the present data in understanding intercellular communication in follicular cells. These results should be helpful for a deeper exploration of the molecular mechanisms governing HF growth. 

## Data Availability

All the relevant data are presented. Other data are available from the corresponding author on reasonable request.

## Supplementary Materials

The following supporting information can be downloaded at: https://www.mdpi.com/article/10.3390/cells12121645/s1. Figure S1: Interactive map of transcription factors identified in DPCs and HMCs; Figure S2: Signaling transduction network mediating intercellular crosstalk from HMCs to DPCs using the first strategy; Figure S3: Signaling transduction network mediating autocrine behavior of DPCs using the first strategy; Figure S4: Signaling transduction network mediating autocrine behavior of HMCs using the first strategy; Figure S5: Signaling transduction network mediating autocrine behavior of DPCs using the secondary strategy; Figure S6: Signaling transduction network mediating autocrine behavior of HMCs using the secondary strategy; Figure S7: Signaling transduction network mediating intercellular crosstalk from HMCs to DPCs using the secondary strategy; Table S1: Parameters used in CCCExplorer analysis; Table S2: Primer information; Table S3: Overall information of sequencing data; Table S4: Differentially expressed genes identified in three groups; Table S5: GO and KEGG pathway enrichment analysis of DEGs; Table S6: Functional signature genes of goat DPCs and HMCs; Table S7: Ligand-receptor pairs mediating autocrine or paracrine cellular crosstalk of DPCs and HMCs.

## Abbreviations

ACVR, activin A receptor; APAF1, apoptotic peptidase activating factor 1; ADM, adrenomedulin; ANGPT2, angiopoietin 2; BMP, bone morphogenetic protein; BNIP3, BCL2 interacting protein 3; BACH1, BTB domain and CNC homolog 1; BAMBI, BMP and activin membrane bound inhibitor; CXADR, CXADR Ig-like cell adhesion molecule; CALCRL, calcitonin receptor like receptor; CREB1, cAMP responsive element binding protein 1; CTNNB1, catenin beta 1; DDIAS, DNA damage induced apoptosis suppressor; DNMT, DNA (cytosine-5)-methyltransferase; EGFR, epidermal growth factor receptor; ESR1, estrogen receptor 1;EGR1, early growth response 1; EGFR, epidermal growth factor receptor; EHF, ETS homologous factor; FGF, fibroblast growth factor; FOS, Fos proto-oncogene; GSK-3β, glycogen synthase kinase 3 beta; HIF1A, hypoxia inducible factor 1 subunit alpha; INHBA, inhibin subunit beta A; IL, interleukin; ITGA1, integrin subunit alpha 1; ITGB6, integrin subunit beta 6; KEGG, Kyoto Encylopaedia of Genes and Genomes; LEP, leptin; LEPR, leptin receptor; LRP6, LDL receptor related protein 6; MCL1, MCL1 apoptosis regulator, BCL2 family member; MMP13, matrix metallopeptidase 13; MAGI3, membrane associated guanylate kinase, WW and PDZ domain containing 3; NFKB1, nuclear factor kappa B subunit 1; NAIP, NLR family apoptosis inhibitory protein; PI3K, phosphatidylinositol 3-kinase; PGF, placental growth factor; PTGR1, prostaglandin reductase 1; PTGES, prostaglandin E synthase; PTGFR, prostaglandin F receptor; PTGFRN, prostaglandin F2 receptor inhibitor; PERP, p53 apoptosis effector related to PMP22; PTCH1, patched 1; RSPO1, R-spondin 1; RORA, RAR related orphan receptor A; REL, REL proto-oncogene, NF-kB subunit; RELA, RELA proto-oncogene, NF-kB subunit; SMAD3, SMAD family member 3; SHH, sonic hedgehog signaling molecule; STAT3, signal transducer and activator of transcription 3; TET, Ten-Eleven Translocation (TET) family protein; VEGFA, vascular endothelial growth factor A; WNT5A, Wnt family member 5A; XIAP, X-linked inhibitor of apoptosis.
